# Supplementary material for: Dynamic transcriptomic profiles of zebrafish gills in response to zinc supplementation
Source: BMC Genomics. 2010 Oct 11;11:553. doi: 10.1186/1471-2164-11-553 (PMC3091702; doi:10.1186/1471-2164-11-553)
Supplement: Additional file 2 — Interactive Direct Interaction Network representing the molecular interactions between zinc, copper, iron, calcium and proteins encoded by transcripts changed by zinc supplementation. Mini web-site containing index.html and hyperlinked pages in subdirectory describing a Direct Interaction Network automatically generated based on curated interactions contained within the proprietary PathwayArchitect database. Ovals represent proteins and the circles symbolize metal ions. Objects are coloured by their abundance in zebrafish at the time-point they were significantly different from the control is a scale from -4 fold (dark green) to +4 fold (dark red). Where significant differences were found at more than one time-point, the colour overlay shows expression at the first instance. Dark blue squares denote 'binding', and light blue squares 'expression'; green squares stand for 'regulation', green diamonds for 'metabolism', and green circles for 'promoter binding'. Arrow heads indicate directionality of the interaction where annotated. All nodes and edges can be further interrogated by selecting the relative area of the image. [file 1471-2164-11-553-S2.zip › PathwayArchitect Zn xs DIN/400263.html]

# BINDING:

|  |  |
| --- | --- |
| Type | BINDING |
| Effect | None |


---

|  |  |
| --- | --- |
| Score | 0 |


---

|  |  |
| --- | --- |
| Reference Count | 32 |


---

|  |  |
| --- | --- |
| Mechanism | Unknown |


---

|  |  |
| --- | --- |
| Reference:0 || Sentence | "Binding of copper to metallothionein provides a temporary storage for cytoplasmic copper, preventing it from occurring as (potentially toxic) free ionic metal." |
| PMID | 8615368 |
| Year | 1996 |
| Species | Human |
| Journal | Am J Clin Nutr |
| RefScore | 0 |
| Source | PArchNLP |
  |
|  |  |


---

|  |  |
| --- | --- |
 Reference:1 || Sentence | "Most of copper present in rat plasma and liver binds to caeruloplasmin and metallothionein, respectively, and is not redox active." |
| PMID | 9702524 |
| Year | 1998 |
| Species | Rat |
| Journal | Free Radic Res |
| RefScore | 1 |
| Source | PArchNLP |
  ||  |  |


---

|  |  |
| --- | --- |
 Reference:2 || Sentence | "Maternal and fetal tissues were assessed for copper concentrations, the activities of the cuproenzymes copper, zinc superoxide dismutase and ceruloplasmin, and the copper binding protein metallothionein." |
| PMID | 8118109 |
| Year | 1993 |
| Species | Rat |
| Journal | Reprod Toxicol |
| RefScore | 1 |
| Source | PArchNLP |
  ||  |  |


---

|  |  |
| --- | --- |
 Reference:3 || Sentence | "Metallothionein is a low molecular mass protein inducible mainly by heavy metals, having high affinity for binding cadmium, zinc and copper." |
| PMID | 7857203 |
| Year | 1994 |
| Species | Rat |
| Journal | Arch Toxicol |
| RefScore | 0 |
| Source | PArchNLP |
  ||  |  |


---

|  |  |
| --- | --- |
 Reference:4 || Sentence | "With larger intakes of zinc, MT synthesis was induced, and the larger amount of this protein was not as readily saturated, resulting in less copper binding to the HMWPF and more to MT." |
| PMID | 6822916 |
| Year | 1983 |
| Species | Rat |
| Journal | J Nutr |
| RefScore | 1 |
| Source | PArchNLP |
  ||  |  |


---

|  |  |
| --- | --- |
 Reference:5 || Sentence | "Metallothionein is a cysteine-rich, low molecular weight protein that binds zinc, copper and cadmium." |
| PMID | 6413769 |
| Year | 1983 |
| Species | Rat |
|  | Human |
| Journal | J Inherit Metab Dis |
| RefScore | 0 |
| Source | PArchNLP |
  ||  |  |


---

|  |  |
| --- | --- |
 Reference:6 || Sentence | "Metallothionein (MT) is a cysteine-rich, low molecular weight protein that binds zinc, copper, and cadmium." |
| PMID | 8236816 |
| Year | 1993 |
| Species | Human |
| Journal | Virchows Arch A Pathol Anat Histopathol |
| RefScore | 1 |
| Source | PArchNLP |
  ||  |  |


---

|  |  |
| --- | --- |
 Reference:7 || Sentence | "Copper binding metallothionein (MT) was induced in a dose-dependent manner and detected at 12 h to 3 days post-instillation." |
| PMID | 8368941 |
| Year | 1993 |
| Species | Rat |
| Journal | Arch Toxicol |
| RefScore | 0 |
| Source | PArchNLP |
  ||  |  |


---

|  |  |
| --- | --- |
 Reference:8 || Sentence | "In contrast, thiomolybdates were much more effective in eliminating exogenous copper and even removed copper that was already bound to metallothionein; thus, the copper level in the heat-stable cytosol fraction decreased." |
| PMID | 1593220 |
| Year | 1992 |
| Species | Rat |
|  | Human |
| Journal | J Lab Clin Med |
| RefScore | 1 |
| Source | PArchNLP |
  ||  |  |


---

|  |  |
| --- | --- |
 Reference:9 || Sentence | "To assay metallothioneins and copper, cadmium, and zinc bound to metallothioneins, we used high-performance liquid chromatography directly coupled to flame atomic absorption spectrometry." |
| PMID | 1497498 |
| Year | 1992 |
| Species | Human |
| Journal | Arch Neurol |
| RefScore | 1 |
| Source | PArchNLP |
  ||  |  |


---

|  |  |
| --- | --- |
 Reference:10 || Sentence | "The pathogenesis of Menkes disease seems to be linked to metallothionein which binds to copper trapped within cells in some tissues." |
| PMID | 4083390 |
| Year | 1985 |
| Species | Human |
| Journal | Brain Dev |
| RefScore | 1 |
| Source | PArchNLP |
  ||  |  |


---

|  |  |
| --- | --- |
 Reference:11 || Sentence | "Vitamin C may prevent the binding of copper and metallothionein by its reducing effect, and excess copper would be released from the cells." |
| PMID | 4083390 |
| Year | 1985 |
| Species | Human |
| Journal | Brain Dev |
| RefScore | 2 |
| Source | PArchNLP |
  ||  |  |


---

|  |  |
| --- | --- |
 Reference:12 || Sentence | "In contrast, metallothionein and caeruloplasmin, proteins specialised to bind copper in vivo had no stimulatory effects." |
| PMID | 2289696 |
| Year | 1990 |
| Species | Human |
| Journal | Free Radic Res Commun |
| RefScore | 2 |
| Source | PArchNLP |
  ||  |  |


---

|  |  |
| --- | --- |
 Reference:13 || Sentence | "Metallothioneins that bind copper and zinc have an Mr of 6500 daltons, consist of a single polypeptide chain of 61 amino acids, 25-30 percent of whose residues are cysteine, have a metal-binding capacity of between 5 and 7 g atoms/mol, and contain no disulfide bonds or aromatic amino acids." |
| PMID | 3283743 |
| Year | 1988 |
| Species | Human |
| Journal | Prog Clin Biol Res |
| RefScore | 2 |
| Source | PArchNLP |
  ||  |  |


---

|  |  |
| --- | --- |
 Reference:14 || Sentence | "Copper-binding MT was induced in the lung tissue following Cu instillation and the amount of MT increased with the dose of CuSO4." |
| PMID | 2267662 |
| Year | 1990 |
| Species | Rat |
| Journal | Toxicology |
| RefScore | 1 |
| Source | PArchNLP |
  ||  |  |


---

|  |  |
| --- | --- |
 Reference:15 || Sentence | "Because of its low copper excretion and reduced binding of copper to metallothionein the Fischer rat, compared to other strains, may be a suitable model for studying the involvement of the liver in copper intoxication." |
| PMID | 3732995 |
| Year | 1986 |
| Species | Rat |
| Journal | Hepatology |
| RefScore | 1 |
| Source | PArchNLP |
  ||  |  |


---

|  |  |
| --- | --- |
 Reference:16 || Sentence | "Following entry, detoxification mechanisms found across species include the binding of copper to specific proteins (e.g. metallothioneins) and the transfer of copper into isolated cell compartments (e.g. periplasmic space, lysosome)." |
| PMID | 8527222 |
| Year | 1995 |
| Species | Human |
| Journal | Annu Rev Nutr |
| RefScore | 1 |
| Source | PArchNLP |
  ||  |  |


---

|  |  |
| --- | --- |
 Reference:17 || Sentence | "Metallothioneins are small, cysteine-rich proteins that avidly bind heavy metals such as zinc, copper, and cadmium to reduce their concentration to a physiological or nontoxic level." |
| PMID | 14612393 |
| Year | 2003 |
| Species | Human |
| Journal | Mol Cell Biol |
| RefScore | 0 |
| Source | PArchNLP |
  ||  |  |


---

|  |  |
| --- | --- |
 Reference:18 || Sentence | "This is explained by the preferential binding of cadmium or copper to metallothionein or its oxidation by H2O2; the concomitant release of zinc in turn leads to the activation of transcription factor MTF-1." |
| PMID | 14612393 |
| Year | 2003 |
| Species | Human |
| Journal | Mol Cell Biol |
| RefScore | 0 |
| Source | PArchNLP |
  ||  |  |


---

|  |  |
| --- | --- |
 Reference:19 || Sentence | "In affected cells, copper significantly accumulates as metallothionein-bound copper in the cytosol and copper transport to the organelles, as well as copper efflux, is disturbed." |
| PMID | 10453200 |
| Year | 1999 |
| Species | Mouse |
|  | Human |
| Journal | Pediatr Int |
| RefScore | 1 |
| Source | PArchNLP |
  ||  |  |


---

|  |  |
| --- | --- |
 Reference:20 || Sentence | "Metallothioneins (MTs) are small cysteine-rich proteins that bind heavy metal ions such as zinc, cadmium and copper with high affinity, and have been functionally implicated in heavy metal detoxification and radical scavenging." |
| PMID | 8065932 |
| Year | 1994 |
| Species | Mouse |
|  | Human |
| Journal | Nucleic Acids Res |
| RefScore | 0 |
| Source | PArchNLP |
  ||  |  |


---

|  |  |
| --- | --- |
 Reference:21 || Sentence | "Metallothioneins (MTs) are well-characterized low molecular weight, heat-stable cytosolic proteins with exceptional high content of cysteinyl sulfur and are known to bind heavy metals like cadmium (Cd), zinc (Zn), and copper (Cu)." |
| PMID | 3293923 |
| Year | 1988 |
| Species | Human |
| Journal | Crit Rev Food Sci Nutr |
| RefScore | 2 |
| Source | PArchNLP |
  ||  |  |


---

|  |  |
| --- | --- |
 Reference:22 || Sentence | "Chromatographic properties of copper-binding proteins (copper-containing metallothioneins) induced in rat liver and kidney by the injection of either copper in the liver and kidney or cadmium in the kidney were compared with those of metallothioneins, with low or no copper content, induced by the injection of cadmium or zinc in the liver both on a Sephadex G-75 column and on a DEAE Sephadex A-25 column." |
| PMID | 485216 |
| Year | 1979 |
| Species | Rat |
| Journal | Arch Environ Contam Toxicol |
| RefScore | 0 |
| Source | PArchNLP |
  ||  |  |


---

|  |  |
| --- | --- |
 Reference:23 || Sentence | "Chromatographical patterns on Sephadex G-75 showed that most of the accumulated copper in the liver and kidney was bound to metallothionein (MT), a low molecular weight protein with high metal binding capacity which plays a role in the detoxification of heavy metals, while no copper bound to MT was found in the lung." |
| PMID | 3192135 |
| Year | 1988 |
| Species | Human |
| Journal | Forensic Sci Int |
| RefScore | 1 |
| Source | PArchNLP |
  ||  |  |


---

|  |  |
| --- | --- |
 Reference:24 || Sentence | "High dietary zinc increased metallothionein-bound copper but did not change the intracellular copper concentration." |
| PMID | 3968585 |
| Year | 1985 |
| Species | Rat |
| Journal | J Nutr |
| RefScore | 0 |
| Source | PArchNLP |
  ||  |  |


---

|  |  |
| --- | --- |
 Reference:25 || Sentence | "Copper pre-loaded in the cells was mostly bound to metallothionein and the amount of copper-metallothionein decreased with time of culture." |
| PMID | 4004887 |
| Year | 1985 |
| Species | Rat |
| Journal | Biochem Pharmacol |
| RefScore | 1 |
| Source | PArchNLP |
  ||  |  |


---

|  |  |
| --- | --- |
 Reference:26 || Sentence | The result showed that liver and kidney induced large amount of metallothioneins that was found to bind to mercury, copper and zinc after mercury intake in stomach and intestines. |
| Year | 2005 |
| PMID | 16395915 |
| Journal | Guang Pu Xue Yu Guang Pu Fen Xi |
| RefScore | 0 |
| Source | PArchNLP |
  ||  |  |


---

|  |  |
| --- | --- |
 Reference:27 || Sentence | This form of copper is toxic, whereas accumulated copper and copper that is bound to ceruloplasmin or metallothionein is not. |
| PMID | 16466879 |
| Year | 2006 |
| Species | Human |
| Journal | Brain Dev |
| RefScore | 1 |
| Source | PArchNLP |
  ||  |  |


---

|  |  |
| --- | --- |
 Reference:28 || Sentence | Zinc induces metallothionein, a highly effective detoxification protein that binds copper. |
| PMID | 16466879 |
| Year | 2006 |
| Species | Human |
| Journal | Brain Dev |
| RefScore | 1 |
| Source | PArchNLP |
  ||  |  |


---

|  |  |
| --- | --- |
 Reference:29 || Sentence | Oral zinc therapy leads to storage of metallothionein-bound copper in the mucosa of the gut and to the excretion of copper via the stools. |
| PMID | 16466879 |
| Year | 2006 |
| Species | Human |
| Journal | Brain Dev |
| RefScore | 1 |
| Source | PArchNLP |
  ||  |  |


---

|  |  |
| --- | --- |
 Reference:30 || Sentence | This indicates that an in vivo metallothionein-copper complex forms the basis of this luminescence. |
| PMID | 16508004 |
| Year | 2006 |
| Journal | Mol Cell Biol |
| RefScore | 0 |
| Source | UserNLP |
  ||  |  |


---

|  |  |
| --- | --- |
 Reference:31 || Sentence | Metallothioneins are small, cysteine-rich proteins that avidly bind heavy metals such as zinc, copper, and cadmium to reduce their concentration to a physiological or nontoxic level. |
| PMID | 14612393 |
| Year | 2003 |
| Species | Human |
| Journal | Mol Cell Biol |
| RefScore | 0 |
| Source | UserNLP |
  |


---

|  |  |
| --- | --- |
